# Epidemiological Characteristics and Prognostic Factors for Mortality in Severe Burns: A 32‐Year Analysis (1993–2024) From a National Referral Center in the Dominican Republic

**DOI:** 10.1002/wjs.70358

**Published:** 2026-04-03

**Authors:** José Enrique Cueva‐Ramírez, Rosario Valdez, Rosy Tejada, Gregorio Gonzalez‐Alcaide, Jose‐Manuel Ramos‐Rincon

**Affiliations:** ^1^ School of Morphological Sciences Faculty of Health Sciences Autonomous University of Santo Domingo Santo Domingo Dominican Republic; ^2^ Pearl F. Ort Burn Unit Santo Domingo Dominican Republic; ^3^ Department of History of Science and Documentation University of Valencia Valencia Spain; ^4^ Clinical Medicine Department University Miguel Hernández of Elche Alicante Spain; ^5^ Internal Medicine Department Dr. Balmis General University Hospital Alicante Spain; ^6^ Alicante Institute for Health and Biomedical Research (ISABIAL) Alicante Spain

**Keywords:** burns, Dominican Republic, epidemiology, mortality, prognosis

## Abstract

**Background:**

Severe burns represent a major public health burden in middle‐income countries. This study describes the epidemiological profile and identifies independent prognostic factors for in‐hospital mortality in patients with severe burns treated at the national referral center of the Dominican Republic over a 32‐year period.

**Methods:**

A retrospective cohort study was conducted including 5941 adult patients with severe burns admitted between 1993 and 2024. Epidemiological, clinical, and outcome data were analyzed. Independent predictors of mortality were identified using multivariable logistic regression.

**Results:**

The cohort was predominantly male (73.5%) with a median age of 34 years. The most common etiology was flame burns (58.2%). The median total body surface area (TBSA) burned was 25%. The overall mortality rate was 28.2%, remaining stable across four eight‐year periods. In the adjusted analysis, the strongest independent predictors of mortality were TBSA ≥ 40% (adjusted odds ratio [aOR] 12.00), age ≥ 65 years (aOR 6.18), the presence of a full‐thickness (third‐degree) component (aOR 2.69), and female sex (aOR 1.23).

**Conclusion:**

Mortality from severe burns remains high and stable over time, driven predominantly by burn extent, depth, and patient age. These findings underscore the critical need to strengthen prehospital care systems and target prevention strategies toward domestic flame and scald risks in similar settings.

## Graphical abstract

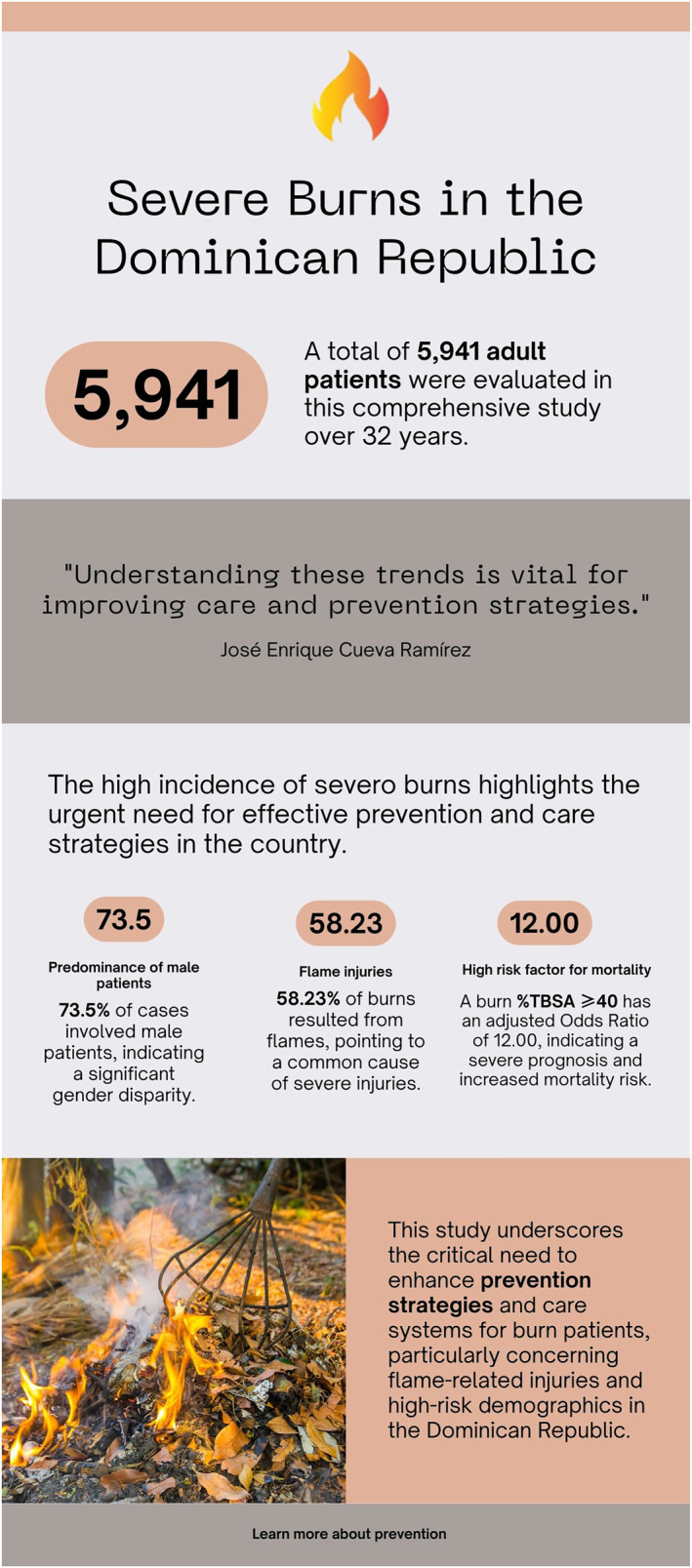

## 1 Introduction

Burn injuries represent a major global public health problem due to their high incidence, significant morbidity, and potential mortality [1, 2]. The World Health Organization estimates that approximately 180,000 people die from burns each year worldwide [2, 3]. These injuries can lead to severe complications such as infection, sepsis, and organ failure [4, 5], as well as long‐term sequelae that impair quality of life [6].

The incidence and mortality of thermal injuries are disproportionately higher in low‐ and middle‐income countries (LMICs) compared to high‐income countries (HICs) [7, 8, 9]. In Latin America, burns remain a significant public health issue; for example, in Mexico, they rank among the leading causes of death [3, 4].

Burn epidemiology varies by region, socioeconomic factors, and age [10, 11, 12, 13]. A consistent global pattern is the male predominance in hospital admissions, with studies from various countries reporting figures that range from 61.9% to 70.9% [4, 12, 14, 15, 16]. Young and middle‐aged adults are consistently identified as the highest risk group internationally [11, 15]. However, the incidence among patients aged over 65 years is increasing internationally, reflecting broader demographic aging—a trend also observed in studies from Spain [1].

Etiology shows marked global contrasts. In high‐income countries (HICs) and some Asian regions, scalds are often the most prevalent cause of severe burns, particularly in pediatric populations [12, 17, 18]. Conversely, in adult populations and trauma centers, flame burns consistently predominate as the leading cause of injury [10, 13]. The growing threat of liquefied petroleum gas (LPG) is notable, with burns related to leaks and explosions prompting calls for stricter preventive measures [2, 5, 17].

Epidemiological studies are crucial for understanding etiology, patient characteristics, and outcomes in specific regions [7]. Universally accepted risk factors for mortality include advanced age, total body surface area (TBSA) burned, and inhalation injury [19, 20, 21, 22]. However, management effectiveness and final prognosis are strongly influenced by the local context of care.

In LMICs, adverse prognostic factors are exacerbated by deficiencies along the care pathway, from prehospital care to hospitalization, including limitations in material and human resources [23, 24]. The high frequency of burns from hazardous agents such as LPG and inadequate initial management contribute to poor outcomes [23, 24].

In the Dominican Republic, the national burn referral center manages the vast majority of patients with severe burns. This centralization provides a highly representative case mix of severe burn injuries at the national level, similar to the role of the National Burn Center in Uruguay [25]. The lack of a robust, updated national epidemiological analysis spanning an extended period limits the health system's capacity to develop targeted prevention strategies and optimize resources. A comprehensive retrospective analysis can provide fundamental data for public health planning, accident prevention, and the improvement of clinical protocols. Therefore, this study aimed to describe the epidemiological characteristics and identify independent prognostic factors for mortality in a cohort of adult patients with severe burns treated at the national referral center in the Dominican Republic over a 32‐year period (1993–2024).

## 2 Methods

### 2.1 Study Design and Setting

A retrospective observational cohort study was conducted to analyze the epidemiological characteristics and risk factors in patients admitted for severe burns to the national burn referral center in Santo Domingo, Dominican Republic, from January 1993 to December 2024.

### 2.2 Ethical Approval

The study protocol was approved by the Institutional Ethics Committee (approval date: 7 February 2024). Due to the retrospective design, the requirement for informed consent was waived. All data were anonymized and handled in strict compliance with institutional guidelines and the principles of the Declaration of Helsinki.

### 2.3 Study Population

**FIGURE 1:**
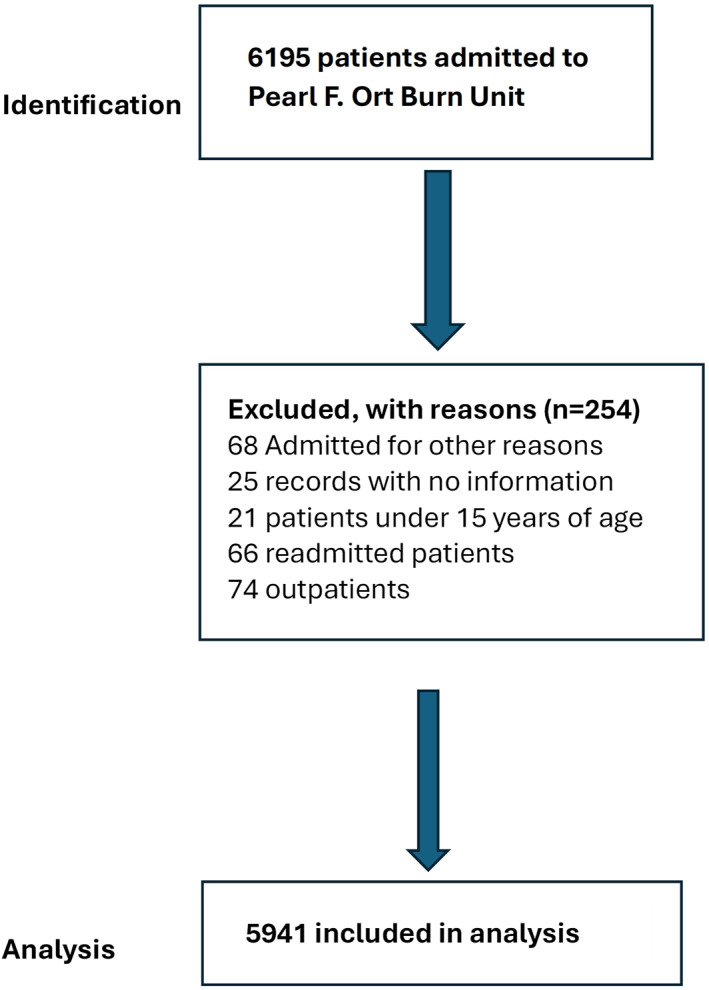
Patient flowchart.

The initial search of institutional archives identified 6195 records. After applying predefined exclusion criteria (age < 15 years, readmissions, incomplete records, and nonburn diagnoses), 254 cases were excluded. The final cohort comprised 5941 adult patients (Figure 1).

### 2.4 Variables and Definitions

The primary outcome was in‐hospital mortality, with the admission rate relative to total emergency visits serving as a secondary outcome. For the purpose of this study, a severe burn was defined by the presence of at least one of the following clinical criteria upon admission: a total body surface area (TBSA) burn of 20% or greater, the presence of full‐thickness (third‐degree) injury, involvement of critical anatomical areas such as the face, hands, feet, perineum, or major joints, or an electrical or chemical etiology, given their associated high risk of complications regardless of surface area.

Explanatory variables encompassed sociodemographic factors like age, sex, nationality, occupation, and geographic origin; epidemiological aspects including injury context, etiology, causal agent, and seasonality; and clinical characteristics such as the percentage of TBSA burned, burn depth, and length of hospital stay. The extent of the burn (TBSA) was clinically estimated by the attending surgeon using the Wallace Rule of Nines. Burn depth and the presence of associated inhalation injury were determined based on the clinical assessment documented in the patient's medical record.

For analysis, key variables were dichotomized using clinically meaningful thresholds. The age cut‐off of 65 years was selected as it is a widely accepted definition of the geriatric population in burn care and is consistently associated with increased morbidity and mortality due to age‐related physiological changes and comorbidities [23, 24]. The TBSA threshold of ≥ 40% was chosen based on its established role as a marker of major severity, often used to define massive burns and trigger specific institutional protocols, and its strong association with poor outcomes in prognostic models [9, 26]. The cut‐off of 19 days for hospital stay was derived from the median length of stay for survivors (12 days) and the upper quartile of the overall cohort, allowing us to differentiate between a “prolonged” stay and the early mortality observed in the most severe, nonsurviving cases. The 32‐year study period was examined in four sequential 8‐year intervals (1993–2000, 2001–2008, 2009–2016, 2017–2024) to assess temporal trends. For the geographical analysis, patients' provinces of origin were aggregated into the 10 standard healthcare regions defined by the Dominican Ministry of Public Health, allowing for the calculation of regional incidence rates based on official population projections.

### 2.5 Statistical Analysis

Statistical analyses were performed using IBM SPSS Statistics version 25.0 (IBM Corp., Armonk, NY, USA) and Epi Info version 7.2.6.0 (Centers for Disease Control and Prevention, Atlanta, GA, USA). Continuous variables are presented as median with interquartile range (IQR) due to nonnormal distribution, and categorical variables as frequencies and percentages.

Group comparisons for temporal trends and sex differences were conducted using the Chi‐square test (or Fisher's exact test for expected cell counts < 5) for categorical variables and the Kruskal–Wallis or Mann–Whitney *U* test for continuous variables, as appropriate.

To identify prognostic factors for in‐hospital mortality, univariable logistic regression was first performed for each candidate variable, calculating crude odds ratios (OR) with 95% confidence intervals (CI). All variables with a *p*‐value < 0.10 in the univariable analysis were considered for inclusion in the multivariable model. A stepwise backward elimination procedure (likelihood ratio test with a removal threshold of *p* > 0.05) was used to construct the final multivariable logistic regression model. The results are presented as adjusted odds ratios (aOR) with 95% CI. The goodness‐of‐fit of the final model was assessed using the Hosmer–Lemeshow test. Multicollinearity among predictors in the final model was evaluated by calculating variance inflation factors (VIF), where a VIF < 5 was considered acceptable.

Missing data were handled by complete‐case analysis for each specific analysis; the number of patients with available data for each variable is reported in the tables (e.g., “*N* contributing data”). A two‐tailed *p*‐value < 0.05 was considered statistically significant.

This manuscript was prepared and edited in accordance with the STROBE (Strengthening the Reporting of Observational Studies in Epidemiology) checklist for cohort studies.

## 3 Results

### 3.1 Cohort Characteristics

**FIGURE 2:**
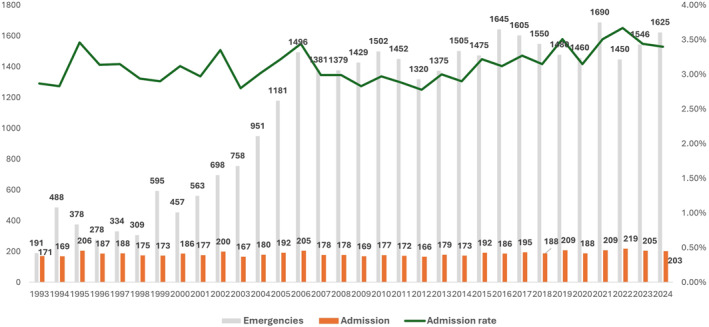
Admissions and emergency consultations by year.

During the study period, 5941 burn patients meeting the selection criteria were admitted, representing 16.7% of the 35,546 burn‐related emergency consultations (Figure 2, Supporting Information S1: Table S1). The cohort had a median age of 34 years (interquartile range [IQR] 25–47) and was predominantly male (73.5%, *n* = 4366). The most affected age groups were 25–44 years (47.7%, *n* = 2821) and 15–24 years (24.3%, *n* = 1437). Dominican nationals comprised 90.1% (*n* = 5335) of the total cohort and the Haitians accounted for 9.5% (*n* = 563) of the cohort (Table 1). Nearly half of the cases (48.4%, *n* = 2755) originated from the Ozama (Metropolitan) Region (Table 1, Supporting Information S1: Table S2). The most frequent occupational sectors were trade, construction, and technical jobs (32.0%, *n* = 1899). A plurality of burns occurred in domestic settings (48.8%, *n* = 1229), followed by occupational settings (33.2%, *n* = 835); acts of aggression accounted for 4.8% (*n* = 121) of cases (Table 1).

**TABLE 1:** Epidemiological and clinical characteristics and patient outcomes in adults admitted for severe burns in the Dominican Republic, 1993–2024.

Variable (*N* contributing data)		*n* (%)^a^
Male sex (*N* = 5941)		4366 (73.5)
Age (years) (*N* = 5941)	Median (IQR)	34 (25–47)
	15–34 years	3090 (52.2)
	35–54 years	1883 (31.8)
	55–74 years	679 (11.5)
	≥ 75 years	266 (4.5)
Nationality (*N* = 5941)	Dominican	5335 (90.1)
	Haitian	563 (9.5)
	Other	43 (0.4)
Region (*N* = 5687)	Ozama Region (metropolitan)	2755 (48.4)
	Valdesia Region	670 (11.8)
	North Cibao Region	462 (8.1)
	Higuamo Region	375 (6.6)
	Yuma Region	344 (6.0)
	Northeast Cibao Region	327 (5.7)
	Other^b^	754 (13.3)
Occupation (*N* = 5941)	Trade, construction, and technical	1895 (31.9)
	Services	988 (16.6)
	Administrative and office staff	851 (14.3)
	Sales and commerce	565 (9.5)
	Unemployed and unspecified	521 (8.8)
	Other^c^	1140 (19.2)
Injury context (*N* = 2515)	Domestic	1229 (48.8)
	Occupational	835 (33.2)
	Assault	121 (4.8)
	Other^d^	330 (13.1)
Etiology (*N* = 5939)	Flame	3458 (58.2)
	Electrical	1688 (28.4)
	Scald	572 (9.6)
	Chemical	175 (2.9)
	Other^e^	46 (0.8)
Causal agent (*N* = 5939)	Gases/fuels	2427 (40.9)
	Electrical current	1830 (30.8)
	Hot liquids	570 (9.6)
	Direct fire	321 (5.4)
	Other^f^	812 (13.7)
TBSA % (*N* = 5913)	Median (IQR)	25 (15–42)
	≥ 40%	1702 (28.8)
Depth (*N* = 5922)	2nd deep, 3rd degree	1998 (33.7)
	2nd superficial, deep	1652 (27.9)
	2nd superficial, deep, 3rd degree	563 (9.5)
	2nd superficial	421 (7.1)
	3rd degree	360 (6.1)
	Other combinations^g^	928 (15.7)
Dichotomized (*N = 5906)*	Without 3rd degree component	2722 (46.1)
	With 3rd degree component	3184 (53.91)
Clinical outcomes (*N* = 5941)	Hospital stay (days), mean, median (IQR)	13.83, 10 (5–19)
	Death	1673/(28.2)

Abbreviations: IQR, interquartile range; NA, Data not available; TBSA, total body surface area.

^a^ Unless otherwise noted.

^b^ Other regions: South Cibao (*n* = 262), Enriquillo (*n* = 214), El Valle (*n* = 201), Northeast Cibao (*n* = 77).

^c^ Other occupation sectors: transportation and logistics (*n* = 403), homemakers/domestic work (*n* = 308), students (*n* = 180), professionals (*n* = 114), administrative and office staff (*n* = 68), agriculture and livestock (*n* = 45), managerial and business (*n* = 22).

^d^ Other injury contexts: public road/street (*n* = 106), electricity theft (*n* = 90), self‐inflicted (*n* = 49), jail/prison (*n* = 25), automobile accident (*n* = 18), shipwreck (*n* = 7), lynching (*n* = 4), not reported (*n* = 31).

^e^ Other causes: contact (*n* = 20), solar radiation (*n* = 7), cold (*n* = 6), not reported (*n* = 13).

^f^ Other causal agents: fires with undefined causes (*n* = 159), acid (*n* = 84), flammable solvents (*n* = 53), hot solids (*n* = 52), industrial radiation/heat (*n* = 16), alkali (*n* = 14), explosives (*n* = 12), various substances (*n* = 12), faulty appliances (*n* = 5), not reported (*n* = 180).

^g^ Other depth(s): 2nd degree (*n* = 358), 1st, 2nd superficial, deep (*n* = 146), 2nd superficial, 3rd (*n* = 126), 1st, 2nd superficial (*n* = 119), 1st, 2nd superficial, deep, 3rd (*n* = 45), 3rd, 4th (*n* = 40), 1st, 2nd deep (*n* = 21), 2nd deep, 3rd, 4th (*n* = 20), 2nd superficial, deep, 3rd, 4th (*n* = 15), NA (*n* = 11), 1st, 2nd deep, 3rd (*n* = 7), 1st (*n* = 5), 4th (*n* = 5), 1st, 2nd superficial, 3rd (*n* = 4), 2nd superficial, deep, 4th (*n* = 1), not reported (*n* = 5).

### 3.2 Temporal and Seasonal Trends

The admission rate relative to total emergency visits showed a marked temporal evolution, peaking at 88.5% in the first year and remaining above 50% for the first six years, before stabilizing at 18.8% or lower from 2004 onward (Supporting Information S1: Table S1). The mean annual number of admissions was 186 (range 169–219), with the maximum recorded in 2022 (*n* = 219). Monthly analysis identified October (*n* = 537), March (*n* = 526), September, and April (*n* = 517 each) as the months with the highest admission frequency (Supporting Information S1: Table S3). Stratifying the 32‐year period into four 8‐year intervals showed that the most recent period (2017–2024) had the highest number of admissions (27.2%, *n* = 1613). Seasonally, the highest proportion of cases occurred in spring (26.2%, *n* = 1555) and the lowest in winter (23.8%, *n* = 1414) (Supporting Information S1: Table S3).

### 3.3 Injury Characteristics and Clinical Outcomes

The primary etiology was flame (58.23%, *n* = 3458), with gases/fuels being the most frequent causal agent (40.9%, *n* = 2427). The median total body surface area burned (TBSA) was 25% (IQR 15–42), with 28.8% of patients (*n* = 1702) presenting extensive burns (TBSA ≥ 40%). Full‐thickness (third‐degree) burns were present in 53.9% of cases (*n* = 3184) (Table 1). The median hospital stay was 10 days (IQR 5–19). The overall in‐hospital mortality rate was 28.2% (*n* = 1673) (Table 1).

### 3.4 Evolution of Patient and Injury Profiles Over Time

Temporal analysis revealed significant shifts in the cohort's profile. The median age increased from 31 to 37 years (*p* < 0.001), and the proportion of patients aged ≥ 65 years rose from 6.6% to 11.3% (*p* < 0.001). The proportion of patients of Dominican nationality decreased from 96.9% to 81.5% (*p* < 0.001). Although flame remained the predominant etiology, its frequency varied significantly (*p* < 0.001), reaching a maximum in the first period (63.7%) and a minimum in the third (53.6%), whereas scalds increased from 8.6% to 12.2% (*p* < 0.001). The median TBSA remained stable at approximately 25%, but the proportion of patients with TBSA ≥ 40% decreased from 30.9% to 26.3% (*p* = 0.001). The presence of a full‐thickness component decreased markedly from 69.5% to 45.1% (*p* = 0.001). Mortality remained stable across periods, ranging from 27.6% to 28.7% (*p* = 0.92) (Table 2).

**TABLE 2:** Epidemiological and clinical characteristics and patient outcomes by study period.

Variables		1st period (1993–2000) (*N* = 1447) *n* (%)^a^	2nd period (2001–2008) (*N* = 1470) *n* (%)^a^	3rd period (2009–2016) (*N* = 1412) *n* (%)^a^	4th period (2017–2024) (*N* = 1612) *n* (%)^a^	*p* value
Male sex		1074 (74.2)	1108 (75.4)	1014 (71.8)	1170 (72.6)	0.12^b^
Age^c^	Median (IQR)	31 (24–42)	33 (24–45)	34 (25–47)	37 (27–5)	< 0.001^d^
	≥ 65 years	95 (6.6)	146 (10.0)	135 (9.6)	182 (11.3)	< 0.001^b^
Dominican nationality		1402 (96.9)	1391 (94.6)	1246 (88.2)	1313 (81.5)	< 0.001^b^
Etiology	Electrical	380 (26.3)	437 (29.7)	416 (29.5)	455 (28.2)	0.15^b^
	Flame	922 (63.7)	898 (61.1)	757 (53.6)	987 (61.2)	< 0.001^b^
	Scald	125 (8.6)	104 (7.1)	147 (10.4)	196 (12.2)	< 0.001^b^
	Chemical	33 (2.30)	35 (2.4)	79 (5.6)	28 (1.7)	< 0.001^b^
	Other	4 (0.30)	5 (0.3)	13 (0.90)	12 (0.7)	0.06^b^
TBSA %	Median (IQR)	25 (15–45)	25 (15–45)	25 (15–40)	24 (15–40)	0.09^d^
	≥ 40%	447 (30.9)	457 (31.1)	402 (28.5)	424 (26.3)	0.001^b^
Depth with 3rd degree component^e^		1005 (69.5)	786 (53.8)	647 (46.2)	697 (45.1)	0.001^b^
Hospital stay^f^	Median days (IQR)	9 (4–18)	10 (5–18)	12 (6–22)	9 (5–17)	< 0.001^d^
	≥ 19 days	355 (24.5)	353 (24.1)	468 (33.3)	380 (23.6)	< 0.001^b^
Death		411 (28.4)	410 (27.9)	390 (27.6)	462 (28.7)	0.92^b^

Abbreviations: IQR, interquartile range; TBSA, total body surface area burned.

^a^ Unless otherwise noted.

^b^ Chi‐square test.

^c^ *N* = 5918.

^d^ Kruskal–Wallis's test.

^e^ *N* = 5906.

^f^ *N* = 5927.

### 3.5 Analysis by Sex

Significant differences were observed between sexes. Women were older (median 35 vs. 33 years, *p* < 0.001) and had nearly three times the odds of being ≥ 65 years (17.0% vs. 6.7%; OR 2.86, 95% CI 2.40–3.42, *p* < 0.001). Mortality was higher in women (32.8% vs. 26.5%; OR 1.35, 95% CI 1.19–1.53, *p* < 0.001). Etiology also differed: electrical burns were less frequent in women (6.9% vs. 36.2%; OR 0.13, 95% CI 0.11–0.16), whereas scalds (17.7% vs. 6.7%; OR 2.97, 95% CI 2.49–3.54) and chemical burns (5.7% vs. 2.0%; OR 2.98, 95% CI 2.20–4.03) were more frequent. Flame was the leading etiology in both sexes but more prevalent in women (68.8% vs. 56.8%; OR 1.67, 95% CI 1.48–1.89). Median TBSA was identical (25%), but women were less likely to have full‐thickness burns (47.5% vs. 55.1%; OR 0.74, 95% CI 0.65–0.83) (Table 3).

**TABLE 3:** Epidemiological and clinical characteristics and patient outcomes by sex.

Variables		Men	Women	OR (CI 95%)	*p* value
Age in years^a^	Median (IQR)	33 (25–45)	35 (24–52)	1.01 (1.01–1.02)	< 0.001^b^
	≥ 65 years, *n* (%)	291 (6.7)	267 (17.0)	2.86 (2.40–3.42)	< 0.001^c^
Dominican nationality, *n* (%)		3946 (90.4)	1406 (89.3)	1.29 (0.93–1.34)	0.21^c^
Etiology, *n* (%)	Electrical	1579 (36.2)	109 (6.9)	0.13 (0.11–0.16)	< 0.001^c^
	Flame	2841 (56.8)	1083 (68.8)	1.67 (1.48–1.89)	< 0.001^c^
	Scalding	294 (6.7)	278 (17.7)	2.97 (2.49–3.54)	< 0.001^c^
	Chemical	86 (2.0)	99 (5.7)	2.98 (2.20–4.03)	< 0.001^c^
	Other	17 (0.4)	17 (1.1)	2.79 (1.42–5.48)	< 0.002^c^
TBSA %	Median (IQR)	25 (15–42)	25 (15–40)	0.99 (0.99–1.00)	0.15^b^
	≥ 40%, *n* (%)	1281 (29.3)	449 (28.5)	0.96 (0.85–1.09)	0.53^c^
Depth with a 3rd degree component^d^, *n* (%)		2392 (55.1)	743 (47.5)	0.74 (0.65–0.83)	< 0.001^c^
Hospital stay, days^e^	Median (IQR)	10 (5–19)	11 (5–19)	1.00 (1.00–1.00)	0.030^b^
	≥ 19 days, *n* (%)	1134 (26.0)	422 (26.8)	1.04 (0.91–1.19)	0.54^c^
Death, *n* (%)		1157 (26.5)	516 (32.8)	1.35 (1.19–1.53)	< 0.001^c^

Abbreviations: CI, confidence interval; IQR, interquartile range; OR, Odds ratio; TBSA, total body surface area burned.

^a^ *N* = 5918.

^b^ Mann‐Whitney *U* test.

^c^ Chi‐square test.

^d^ *N* = 5906.

^e^ *N* = 5927.

### 3.6 Risk Factors for In‐Hospital Mortality

Patients who died were significantly older than survivors (median 40 vs. 32 years, *p* < 0.001). The final multivariable model included age, sex, burn etiology, TBSA, and burn depth. Age ≥ 65 years was associated with 6.18‐fold increased odds of death (aOR 6.18, 95% CI 4.85–7.70, *p* < 0.001), whereas female sex was an independent risk factor (aOR 1.23, 95% CI 1.05–1.47, *p* = 0.01). TBSA was markedly higher in deceased patients (median 50% vs. 20%, *p* < 0.001), and TBSA ≥ 40% showed the strongest association with mortality (aOR 12.00, 95% CI 10.30–13.98, *p* < 0.001). The presence of a full‐thickness burn was also a significant independent predictor (aOR 2.69, 95% CI 2.65–3.13, *p* < 0.001). Flame etiology showed a strong association in univariable analysis, whereas electrical burns were associated with lower mortality (aOR 0.49, 95% CI 0.29–0.86, *p* = 0.013). Hospital stay was significantly shorter in deceased patients (median 6 vs. 12 days, *p* < 0.001) (Table 4).

**TABLE 4:** Risk factors for in‐hospital mortality in patients admitted for severe burns.

Variables		Survival (*N* = 4249)	Death (*N* = 1669)	Crude analysis		Adjusted analysis^a^	
				OR (95% CI)	*p* value	OR (95% CI)	*p* value
Age^b^	Median (IQR)	32 (24–43)	40 (28–58)	1.03 (1.02–1.03)	< 0.001^c^	NI^d^	
	≥ 65 years	247 (5.8)	311 (18.6)	3.71 (3.11–4.43)	< 0.001^e^	6.18 (4.85–7.70)	< 0.001
Female sex		1059 (24.8)	516 (30.8)	1.35 (1.19–1.53)	< 0.001^e^	1.23 (1.05–1.47)	0.01
Dominican nationality		3841 (90.0)	1511 (90.3)	0.96 (0.8–1.17)	0.13^e^	—	
Period	1993–2000	1036 (24.3)	411 (24.6)	1		—	
	2001–2008	1060 (24.8)	410 (24.5)	0.97 (0.83–1.15)	0.76^e^	—	
	2009–2016	1022 (23.9)	390 (23.3)	0.96 (0.82–1.13)	0.64^e^	—	
	2017–2024	1150 (26.9)	462 (27.6)	1.10 (0.86–1.19)	0.87^e^	—	
Etiology	Electrical	1405 (32.9)	283 (16.9)	0.42 (0.36–0.48)	< 0.001^e^	0.49 (0.29–0.86)	0.013
	Flame	2252 (52.8)	1312 (78.4)	3.25 (2.85–3.71)	< 0.001^e^	0.98 (0.56–1.73)	0.96
	Scald	501 (11.7)	71 (4.2)	0.33 (0.25–0.43)	< 0.001	0.58 (0.31–1.09)	0.09
	Chemical	152 (3.6)	23 (1.4)	0.38 (0.24–0.59)	< 0.001^e^	0.55 (0.26–1.17)	0.12
	Other	31 (0.7)	3 (0.2)	0.25 (0.08–0.80)	0.012	0.17 (0.04–0.92)	0.04
TBSA %	Median (IQR)	20 (12–30)	50 (33–73)	1.07 (1.07–1.08)	< 0.001^c^	NI^d^	
	≥ 40%	601 (14.1)	1129 (67.5)	12.66 (11.08–14.48)	< 0.001	12.00 (10.30–13.98)	< 0.001
Depth with 3rd degree component^f^		1921 (45.4)	1214 (74.2)	3.19 (2.82–3.61)	< 0.001^e^	2.69 (2.65–3.13)	< 0.001
Hospital stay (days)^g^	Median (IQR)	12 (6–21)	6 (3–13)	0.96 (0.95–0.97)	< 0.001^c^	NI^d^	
	≥ 19 days	1288 (30.3)	268 (16.0)	0.43 (0.38–0.51)	< 0.001^e^	NI^d^	

Abbreviations: CI, confidence interval; IQR, interquartile range; OR, odds ratio.

^a^ Continuous variables (age, TBSA, hospital stay) were not included in the multivariate analysis.

^b^ *N* = 5918 for age.

^c^ Mann‐Whitney *U* test.

^d^ NI = Not included (these are continuous variables; only categorical variables were included in the model).

^e^ Chi‐square test.

^f^ *N* = 5906 for depth.

^g^ *N* = 5927 for hospital stay.

## 4 Discussion

This 32‐year analysis of 5941 patients with severe burns provides fundamental data on the clinical and epidemiological burden of this injury in a middle‐income Caribbean nation. The admission rate of 16.7% aligns with the proportion of severe cases requiring inpatient management reported in other settings [8].

The evolution of the admission‐to‐consultation ratio, which decreased markedly from 88.5% in 1993 to 18.8% or less from 2004 onward, warrants careful interpretation within the context of a single national referral center in a middle‐income country.

Contrary to what might be inferred as the implementation of a formal triage system, this trend is best explained by a progressive overload of the emergency department with consultations for minor burns. As the only specialized center for adult burns in the Dominican Republic, our institution became the default recipient for all burn‐related referrals from hospitals across the public health network. Over time, peripheral hospitals increasingly adopted the practice of transferring any patient with a burn injury, regardless of severity, citing the “lack of a burn unit” as the primary reason for referral. This behavior, likely driven by pressures to optimize local bed capacity and budgets, resulted in a steady and substantial increase in the volume of emergency consultations for minor burns (the denominator of the ratio).

Meanwhile, the absolute number of admissions for severe burns (the numerator) remained relatively stable over the study period, as shown in Figure 2. Consequently, the sharp decline in the admission‐to‐consultation ratio reflects a dilution effect caused by the influx of minor cases, rather than a more selective admission policy for severe cases. This finding highlights a critical challenge faced by reference centers in resource‐limited settings: they often absorb the full burden of a pathology from the entire healthcare network, which can strain emergency services and obscure the true epidemiology of severe disease. The stabilization of the ratio at around 18% after 2004 likely represents the point at which the center reached a steady state, absorbing all minor burns referred nationally while maintaining a constant capacity to admit the most severe cases.

The relatively higher volume of admissions in the most recent period (27.2% of the total) is consistent with a high cumulative incidence relative to the population reported in Spain [1]. Seasonal analysis revealed the highest proportion of cases in spring (26.2%), particularly in March and April, followed by autumn (25.8%). This pattern differs from peaks reported in other regions [21] highlighting regional climatic and behavioral variations. The high incidence of burns caused by gases/fuels (40.7%) mirrors patterns of liquefied petroleum gas (LPG) use and related risks reported in others countries [17]. The overall mortality rate of 28.2% reflects the high severity of cases managed at this national referral center and is comparable to rates reported in other middle‐income countries [2, 7], while contrasting sharply with figures below 5% often observed in high‐income settings [1].

The median TBSA of 25% confirms the high severity of the cohort, exceeding figures reported in general burn unit populations from other countries [1, 9], consistent with the center's role in centralizing the most severe cases. The male predominance (73.5%) aligns with global patterns consistently reported in the literature [16, 22]. A notable finding was the significant aging of the cohort over 32 years, with the median age increasing from 31 to 37 years and the proportion of patients aged ≥ 65 years doubling from 6.6% to 11.3%. This demographic shift parallels global trends and underscores the need for adapted clinical management strategies for older burn victims.

Flame was the primary etiology (58.2%), largely driven by gases/fuels (40.7%), a pattern closely linked to the widespread domestic use of LPG in individual tanks, often installed through nonprofessional connections. This aligns with reports from other middle‐income countries where flame is the predominant etiology [2, 22]. This pattern is closely linked to the widespread domestic use of liquefied petroleum gas (LPG) in individual tanks, a risk factor well‐documented in the literature [17]. Scalds (9.6%) were more frequent in women, contrasting with their predominance in pediatric populations in high‐income countries [3].

In the multivariable analysis, TBSA ≥ 40% was the strongest predictor of mortality (aOR 12.00), reaffirming the universal prognostic centrality of burn extent [26]. Age ≥ 65 years was also a powerful independent risk factor (aOR 6.18), corroborating advanced age as a key determinant of poor outcomes [4]. The presence of full‐thickness burns was a significant predictor (aOR 2.69), supporting clinical protocols that prioritize referral of these injuries [6]. Notably, female sex emerged as an independent risk factor for death (aOR 1.23). In contrast to contexts where self‐immolation is a prominent factor [27], this association in our cohort likely reflects distinct local determinants, such as differential exposure to domestic accidents or potential delays in care‐seeking.

The shorter hospital stay among deceased patients (median 6 vs. 12 days) suggests early mortality in the most catastrophic cases, a pattern consistent with other studies [22].

The relatively short median hospital stay of 10 days (12 days for survivors), despite a high median TBSA of 25% and a predominance of full‐thickness burns, warrants specific explanation. This finding likely reflects a combination of factors characteristic of the healthcare context in many middle‐income countries. First, it may be influenced by a high early mortality rate (median stay of 6 days in deceased patients), which truncates the overall length of stay for the most severe cases. Second, and more importantly, it may reflect systemic limitations in the capacity to provide prolonged, specialized multidisciplinary care, including multiple surgical interventions for grafting and prolonged rehabilitation. Resource constraints, such as limited operating room availability, shortages of critical care beds, or challenges in providing complex nutritional support, could lead to a tendency for less aggressive surgical management or earlier discharge to sub‐acute facilities or home care, particularly when the prognosis is perceived as poor or when beds are needed for new admissions. This pattern underscores that length of stay is not solely a marker of injury severity but also is heavily modulated by local healthcare system capacity and clinical practices, a crucial consideration when comparing outcomes internationally.

### 4.1 Strengths and Limitations

The principal strength of this study lies in its unprecedented 32‐year longitudinal design and large national cohort (*N* = 5941), providing high statistical power and a comprehensive view of severe burn epidemiology. The findings are highly representative of severe cases in a middle‐income country, as evidenced by the concentration of critical injuries (median TBSA 25%) at the nation's sole referral center. Methodological rigor was ensured through multivariable logistic regression and detailed temporal and geographical stratification.

The main limitation is inherent selection bias; whereas centralization ensures representativeness for severe burns, findings may not generalize to less severe injuries managed elsewhere. The retrospective design over 32 years carries a risk of documentation inconsistencies. The predictive model's comprehensiveness is limited by the absence of consistently recorded variables such as inhalation injury, a common challenge in retrospective burn studies. This omission, although a limitation, highlights a critical area for improvement in future data collection efforts, such as a prospective registry, to enable more precise risk stratification. Finally, the high mortality rate must be interpreted within the context of documented systemic limitations in prehospital care and resource constraints prevalent in similar settings.

### 4.2 Future Research

These findings highlight several critical research avenues. Furthermore, the independent association of female sex with mortality merits deeper investigation. Future prospective studies should aim to disentangle the potential contributors to this disparity, including analysis of potential differences in pre‐hospital care‐seeking behavior, time from injury to definitive care, physiological response to injury, and the clinical management received (e.g., fluid resuscitation, surgical timing and intensity) between male and female patients.

Prospective studies are also needed to quantify long‐term morbidity, quality of life, and socio‐occupational reintegration of survivors. Applied research should inform targeted prevention programs focusing on domestic scald risks in the elderly, safe LPG use, and occupational safety. Establishing a prospective clinical registry is essential for capturing acute prognostic variables (such as inhalation injury) and validating mortality risk scores locally. Finally, system‐level analysis is required to investigate the impact of prehospital delays and optimize the continuum of care from injury to definitive treatment.

## 5 Conclusion

In this national 32‐year cohort, the prognosis of severe burns is predominantly determined by the extent and depth of the injury and the patient's age, which must remain the cornerstone of initial risk stratification. The persistently high mortality rate, stable over 3 decades, points to critical systemic challenges extending beyond hospital care, underscoring an urgent need to strengthen prehospital response and referral systems. The documented demographic shift toward an older cohort and the predominance of preventable domestic flame injuries call for targeted prevention strategies. These findings highlight the severe burden of burn injuries and underscore the necessity for further research into the long‐term outcomes and rehabilitation of survivors.

## Author Contributions

**Jose Enrique Cueva‐Ramírez:** writing – original draft, writing – review and editing, project administration, supervision, data curation, formal analysis, methodology, validation, investigation, conceptualization, visualization. **Rosario Valdez:** investigation, data curation, conceptualization, formal analysis. **Rosy Tejada:** formal analysis, data curation, validation, investigation. **Gregorio Gonzalez‐Alcaide:** visualization, validation, supervision, data curation, conceptualization. **Jose‐Manuel Ramos‐Rincon:** writing – original draft, investigation, conceptualization, methodology, visualization, writing – review and editing, formal analysis, data curation, supervision, project administration.

## Funding

The authors have nothing to report.

## Ethics Statement

The study protocol was approved by the Hospital Ethics Committee on 7 February 2024. This research was conducted in strict compliance with the hospital's ethical guidelines and applicable national regulations governing biomedical research. Informed consent was obtained from all participants or their legal guardians.

## Conflicts of Interest

The authors declare no conflicts of interest.

## Supporting information


Supporting Information S1

## Data Availability

The datasets generated and analyzed during this study are not publicly available due to patient confidentiality and institutional regulations but may be available from the corresponding author upon reasonable request, subject to approval from the Hospital Ethics Committee.
