# Supplementary material for: Epidemiological Characteristics and Prognostic Factors for Mortality in Severe Burns: A 32‐Year Analysis (1993–2024) From a National Referral Center in the Dominican Republic
Source: World J Surg. 2026 Apr 3;50(5):1159–68. doi: 10.1002/wjs.70358 (PMC13206501; doi:10.1002/wjs.70358)
Supplement: Supplementary file 1 — Supporting Information S1 [file WJS-50-1159-s001.docx]

**Appendix A**

**Supplementary material**

**Table S1.** Hospital emergency consultations and admissions for burn injuries in the Pearl F. Ort Burn Unit, Dominican Republic, 1993-2024

| Year | N admissions | N emergencies for severe burns | Admissions rate (%row) | % admissions over study period (%col) |
| --- | --- | --- | --- | --- |
| 1993 | 169 | 191 | 88.5% | 2.8% |
| 1994 | 169 | 488 | 34.6% | 2.8% |
| 1995 | 205 | 378 | 54.2% | 3.5% |
| 1996 | 187 | 278 | 67.3% | 3.2% |
| 1997 | 185 | 334 | 55.4% | 3.1% |
| 1998 | 175 | 309 | 56.6% | 3.0% |
| 1999 | 172 | 595 | 28.9% | 2.9% |
| 2000 | 185 | 457 | 40.5% | 3.1% |
| 2001 | 177 | 563 | 31.4% | 3.0% |
| 2002 | 200 | 698 | 28.7% | 3.4% |
| 2003 | 164 | 758 | 21.6% | 2.8% |
| 2004 | 179 | 951 | 18.8% | 3.0% |
| 2005 | 190 | 1181 | 16.1% | 3.2% |
| 2006 | 204 | 1496 | 13.6% | 3.4% |
| 2007 | 178 | 1381 | 12.9% | 3.0% |
| 2008 | 178 | 1379 | 12.9% | 3.0% |
| 2009 | 169 | 1429 | 11.8% | 2.8% |
| 2010 | 177 | 1502 | 11.8% | 3.0% |
| 2011 | 171 | 1452 | 11.8% | 2.9% |
| 2012 | 166 | 1320 | 12.6% | 2.8% |
| 2013 | 179 | 1375 | 13.0% | 3.0% |
| 2014 | 173 | 1505 | 11.5% | 2.9% |
| 2015 | 191 | 1475 | 13.0% | 3.2% |
| 2016 | 186 | 1645 | 11.3% | 3.1% |
| 2017 | 193 | 1605 | 12.0% | 3.3% |
| 2018 | 188 | 1550 | 12.1% | 3.2% |
| 2019 | 208 | 1480 | 14.1% | 3.5% |
| 2020 | 187 | 1460 | 12.8% | 3.2% |
| 2021 | 209 | 1690 | 12.4% | 3.5% |
| 2022 | 219 | 1450 | 15.1% | 3.7% |
| 2023 | 205 | 1546 | 13.3% | 3.4% |
| 2024 | 203 | 1625 | 12.5% | 3.4% |
| *Total* | ***5941*** | ***35,546*** | ***16.7%*** | ***100.0%*** |

**Table S2.** Admissions for severe burns and estimated population, by regions of the Dominican Republic and study period

| **Region** | **1st period**  **(1993-2000)** | | **2nd period**  **(2001-2008)** | | **3rd period**  **(2009-2016)** | | **4th period**  **(2017-2024)** | |  |
| --- | --- | --- | --- | --- | --- | --- | --- | --- | --- |
|  | **Cases** | **Population^a^** | **Cases** | **Population** | **Cases** | **Population^a^** | **Cases** | **Population^a^** | **Total** |
| Metropolitan Region | 780 | 3,573,689 | 706 | 2,678,097 | 611 | 3,717,178 | 658 | 4,171,715 | 2755 |
| Northern Cibao | 106 | 3,875,526 | 111 | 4,492,682 | 109 | 5062057 | 136 | 5,580,518 | 462 |
| Southern Cibao | 47 | 690,457 | 44 | 711,017 | 49 | 728,586 | 73 | 744,571 | 213 |
| Northeast Cibao | 43 | 596,169 | 57 | 622,225 | 50 | 637,663 | 69 | 649,468 | 219 |
| Northwest Cibao | 11 | 383,562 | 6 | 394,311 | 21 | 409,325 | 39 | 424,856 | 77 |
| Valdesia | 154 | 955,325 | 185 | 1,019,402 | 162 | 1,080,758 | 156 | 1,139,468 | 657 |
| Enriquillo | 49 | 336,242 | 58 | 364,009 | 46 | 378,385 | 61 | 387,686 | 214 |
| El Valle | 40 | 299,222 | 62 | 297,933 | 49 | 289,933 | 50 | 278,807 | 201 |
| Yuma | 75 | 481,681 | 81 | 582,634 | 85 | 676,460 | 102 | 760,680 | 343 |
| Higuamo | 92 | 558,838 | 196 | 564,311 | 78 | 574,709 | 101 | 587,465 | 467 |
| ***Dominican Republic*** | ***1408*** | ***11,750,711*** | ***1511*** | ***11,726,621*** | ***1380*** | ***13,555,054*** | ***1564*** | ***14,725,234*** | ***5863*** |

^a^ *Source*: <https://www.one.gob.do/media/v1cgwmsp/cuadro-estimaciones-proyecciones-poblaci%C3%B3n-total-por-a%C3%B1o-seg%C3%BAn-regi%C3%B3n-provincia-2000-2030.xlsx>

**Table S3.** Admissions for severe burns by period

| Category | Time interval | Admissions (%) | Total emergencies (%) | Admissions rate |
| --- | --- | --- | --- | --- |
| Study period | Overall: 1993-2024 | 5941 (100) | 35,546 (100) | 16.7% |
|  | 1993-2000 | 1456 (24.5) | 3030 (8.5) | 48.1% |
|  | 2001-2008 | 1458 (24.5) | 8407 (23.7) | 20.0% |
|  | 2009-2016 | 1414 (23.8) | 11,299 (31.8) | 12.5% |
|  | 2017-2024 | 1613 (27.2) | 12,806 (36.0) | 12.6% |
| Season | Spring | 1555 | — | — |
|  | Autumn | 1532 | — | — |
|  | Summer | 1440 | — | — |
|  | Winter | 1414 | — | — |
| Month | October | 537 | — | 9.0% |
|  | March | 526 | — | 8.9% |
|  | April | 517 | — | 8.7% |
|  | September | 517 | — | 8.7% |
|  | May | 512 | — | 8.6% |
|  | August | 504 | — | 8.5% |
|  | December | 489 | — | 8.2% |
|  | November | 478 | — | 8.0% |
|  | July | 476 | — | 8.0% |
|  | January | 473 | — | 8.0% |
|  | June | 460 | — | 7.7% |
|  | February | 452 | — | 7.6% |
| *Total* |  | ***5941*** | ***—*** | ***100.0%*** |
